# F184. TESTING THE GABA-GLUTAMATE HYPOTHESIS FOR SCHIZOPHRENIA IN RELATION TO AUDITORY HALLUCINATIONS - PRELIMINARY RESULTS

**DOI:** 10.1093/schbul/sby017.715

**Published:** 2018-04-01

**Authors:** Helene Hjelmervik, Alexander R Craven, Erik Johnsen, Kristiina Kompus, Rune A Kroken, Else-Marie Løberg, Kenneth Hugdahl

**Affiliations:** 1 University of Bergen; 2 University of Bergen, NORMENT Center of Excellence, University of Oslo; 3 Haukeland University Hospital, NORMENT Center of Excellence, University of Oslo; University of Bergen

## Abstract

**Background:**

The gamma-aminobutyric acid (GABA)-glutamate hypothesis of schizophrenia suggests a neurotransmitter imbalance – reduced GABA and increased glutamate - which causes disruption of the modulation between inhibitory GABAergic interneurons and excitatory glutamatergic neurons. In the left superior temporal gyrus (STG) both hyperactivation and increased glutamate levels have previously been associated with auditory hallucinations in schizophrenia patients. However, the STG GABA-glutamate imbalance by simultaneously measuring GABA and glutamate in the same subjects has not previously been tested, and was therefore the aim of the present study. We hypothesized reduced GABA and increased glutamate in the patients relative to controls. Furthermore, reduced GABA and elevated glutamate in STG should be related to severity of auditory hallucinations in these patients.

**Methods:**

The current study tested 23 schizophrenia patients (18 hallucinating and 5 non-hallucinating) and 53 healthy controls. The sample included both female and male participants above the age of 18. All patients were on medication, and they were tested at different times relative to the treatment onset. Magnetic resonance spectroscopy (MRS) was used to acquire data from voxels in the right and left superior temporal gyrus (Heschl’s gyri) with a 3T GE 750 Discovery MR scanner. PRESS and MEGA-PRESS sequences were applied to measure glutamate and GABA, respectively. Voxel tissue water was used as reference for glutamate and GABA. Scores on the Positive and Negative Syndrome Scale (PANSS) were also collected, and used to differentiate hallucinating from non-hallucinating patients.

**Results:**

Separate 2(Group) x 2(Hemisphere) ANOVAs were estimated for GABA and glutamate. No main or interaction effects came out significant for GABA (All F(1,73)<2.7, p>0.1, η2<0.03). The analysis for glutamate resulted in a main effect of Hemisphere (F(1,74)=24, p<0.001, η2=0.25) in which the right STG showed overall higher concentrations than the left STG. In addition, an interaction effect between Group and Hemisphere was found (F(1,74)=5.22, p=0.03, η2=0.07). Bonferroni Post-hoc analysis showed significantly elevated glutamate levels in patients relative to controls in the right STG only (p=0.005). Furthermore, a multiple regression analysis was estimated between severity of hallucinations (PANSS P3 item) at the time of testing, and GABA and glutamate values in left and right STG. Although the overall model fit was non-significant, an approximate significant correlation was found between hallucination severity and left STG glutamate levels (β=0.36, t=2.1, SE=0.17, p=0.05).

**Discussion:**

The present study found higher glutamate levels in schizophrenia patients relative to healthy controls in the right STG. In spite of no overall differences in glutamate in the left STG, as initially hypothesized, glutamate levels in this region was found to predict severity of auditory hallucinations. One could speculate that the additional neuronal activity associated with auditory hallucinations elevate glutamate to ‘normal levels’ corresponding to that of healthy controls. The increased glutamate levels in the right STG seems (linearly) unrelated to auditory hallucinations and future analysis should test whether other symptoms are related to this finding. Overall, the study found only limited support for the GABA-glutamate hypothesis; In spite of increased glutamate in one of the regions, GABA was not found to be reduced in patients and was unrelated to auditory hallucinations.

